# Opto-Microfluidic Integration of the Bradford Protein Assay in Lithium Niobate Lab-on-a-Chip

**DOI:** 10.3390/s22031144

**Published:** 2022-02-02

**Authors:** Leonardo Zanini, Annamaria Zaltron, Enrico Turato, Riccardo Zamboni, Cinzia Sada

**Affiliations:** 1 Physics and Astronomy Department, University of Padova, Via Marzolo 8, 35131 Padova, Italy; leonardo.zanini.2@phd.unipd.it (L.Z.); enrico.turato@ftf.lth.se (E.T.); 2 Institute of Applied Physics, University of Münster, Corrensstrasse 2/4, 48149 Muenster, Germany; riccardo.zamboni@uni-muenster.de

**Keywords:** Bradford assay, opto-microfluidics, protein quantification

## Abstract

This paper deals with the quantification of proteins by implementing the Bradford protein assay method in a portable opto-microfluidic platform for protein concentrations lower than 1.4 mg/mL. Absorbance is measured by way of optical waveguides integrated to a cross-junction microfluidic circuit on a single lithium niobate substrate. A new protocol is proposed to perform the protein quantification based on the high correlation of the light absorbance at 595 nm, as commonly used in the Bradford method, with the one achieved at 633 nm with a cheap commercially available diode laser. This protocol demonstrates the possibility to quantify proteins by using nL volumes, 1000 times less than the standard technique such as paper-analytical devices. Moreover, it shows a limit of quantification of at least 0.12 mg/mL, which is four times lower than the last literature, as well as a better accuracy (98%). The protein quantification is obtained either by using one single microfluidic droplet as well by performing statistical analysis over ensembles of several thousands of droplets in less than 1 min. The proposed methodology presents the further advantage that the protein solutions can be reused for other investigations and the same pertains to the opto-microfluidic platform.

## 1. Introduction

Among all the biomolecules, proteins play a key role in repairing and maintenance processes of human cells, tissues and organs. They strongly contribute to energy-providing processes as well as hormones regulation and metabolic processes [1,2], participating in regulating metabolic processes as enzymes [3]. Protein levels are routinely measured in clinical settings for diagnosis purposes since they act as indicators to detect inflammations [4], nutritional status diseases [5], dehydration [1], and organ disorders [2,3], just to cite some examples. The measure of the total protein content plays a key role also in controlling the production of biopharmaceuticals, especially if they are synthesized or drug manufactured from biological sources such as cells and cellular components (6 and references therein quoted). Several analytical methods have been implemented to perform accurate protein detection depending on the needed information, such as UV-vis spectrometry, electrophoresis, mass spectrometry, proteomics, immunoblotting [6,7]. These techniques suffer from using a mL liquid volume and require longer preparation time, expensive, bulky instruments for protein detection and specialized expertise to complete the protocols. As alternatives, total protein analysis is carried out by colorimetric protein assays [8,9,10,11], which are based on colorimetric reactions promoted when the protein solution reacts with a specific dye-based reagent. They lead to the formation of a dye-protein complex responsible for shifting the dye absorbance by a detectable amount depending on the protein concentration. This is the case of the well-known Bradford assay where the dye Brilliant Blue G (Coomassie Brilliant Blue G-250) is used and the protein–dye complexes cause a shift in the dye absorption maximum from 465 nm to 595 nm [12]. This is currently done in laboratory conditions that need a transfer of samples from the biotechnology production plant. The Bradford assay is sampled and then measured by optical absorbance techniques or paper-analytical devices (PADs), better known as protein test strips [13], or paper microfluidic platforms [14]. While qualitative and semi-quantitative results are obtained by comparing the assay color in color chart, smartphones or scanners have been also implemented for quantitative results [15,16,17,18]. Despite being unprecise in quantification if compared to proteomics implemented approaches, PADs still remain the favorite method for a first-level check of the production processes. Treated as “golden standard” in any protein-based application [19,20], they have been widely implemented in biopharmaceuticals to “pass/not pass” quality assessment process and to identify batches to be further analyzed with more expensive techniques. PADs are convenient because they are cheaper, easy to handle, portable, and they ensure a lower consumption of samples and reagents per assay with respect to common spectrophotometric methods [14]. Unfortunately, they still present some limitations on the detection limits and average sensitivity. PADs performances have been investigated to some extent in [6], where different assays have been exploited (Biuret [21], Lowry [22,23], Bicinchoninic acid (BCA) [24]), Bradford assay [12], Bromocresol green (BCG) and Tetrabromophenol blue (TBPB) assays [25] respectively, tested on Bovine Serum Albumin (BSA) as protein standard [6]. As far as the Bradford protein assay is concerned, the authors reported on results achieved with BSA dilution series in the range 0.05–4 mg/mL, directly measuring the optical density signals (absorption at 595 nm). By testing several working conditions, they have been able to identify the best performance when using the following parameters: reagent volume of 10 μL of protein solution in microfiber (in the scanned range of 5–10 microliters), 14 min of assay time (investigated range 0–20 min) and reagent concentration equal to 0.01% (investigated range 0.01–0.5%). The calibration curve showed a linear range 0.1–3.0 mg/mL (with a r^2^ = 0.967), an accuracy of 94.1 ± 6.4%, slope of 8.43, precision 3.1%, limit of detection LOD 0.5 mg/mL and limit of quantitation LOQ equal to 1.5 (mg/mL), respectively. For unknown BSA dilution, the measurement result error was estimated to be lower respect the standard method, such as 72.8 µg/mL in chip and 73.4 µg/mL in tube, respectively [6]. These results clearly demonstrate that PADs are of great interest but unsuccessful when nL or pL protein solutions are under investigation. Moreover, they are not suitable for monitoring the protein synthesis protocol especially when the latter is made of several critical steps and a long time of execution.

The availability of efficient, cheaper, and simple methods and/or devices for bioanalysis is therefore of great interest for medicine, biopharma and agriculture for food quality determination applications, especially if portable, fast, easy, using pL and nL of protein solution and with better detection limits respect to the current ones even in microfluidics implemented platforms [26,27,28,29,30,31,32].

This paper deals with the quantification of protein within protein-based solutions with lower detection limits and higher sensitivity by way of a Bradford protein assay method efficiently implemented in a portable opto-microfluidic platform in a protein concentration regime lower than 1.4 mg/mL. It demonstrates the possibility to quantify protein concentration by using one single microfluidic droplet, i.e., the volume of the order of nL or lower, as well as performing statistical analysis over ensembles of several thousands of droplets in less than 1 min. The proposed approach has the further advantage of being able to reuse the analyzed solution for further investigation if needed.

## 2. Materials and Methods

**Figure 1:**
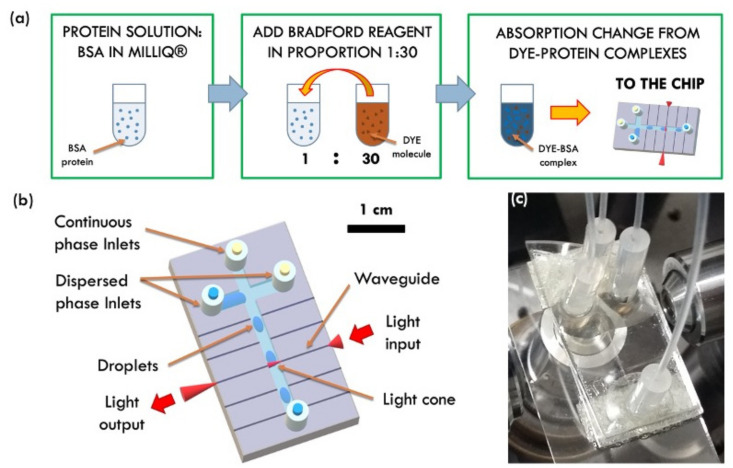
(**a**) Scheme of the reaction system: the protein solution is prepared and then analyzed by the opto-microfluidic platform. (**b**) Sketch of the microfluidic platform: the optical waveguides are normal to the main microfluidic channel where the continuous phase flows. The protein solution is injected by way of the dispersed phase inlets. The microfluidic cross-junction provides the generation of droplets flowing into the main microfluidic channel and is illuminated by the light exiting from the input waveguide. The transmitted light is collected by the output waveguide and then recorded by the detector coupled at its exit. (**c**) Picture of the opto-microfluidic platform.

Different protein concentrations have been prepared in the range 0.14−1.4 mg/mL and compared to the baseline (0 mg/mL, i.e., zero protein reference). The protein used in this work is the Bovine Serum Albumine (BSA, supplier Merck KGaA, Frankfurter Straße 25064293 Darmstadt, Germany), a typical standard protein in biological research. The Bradford reagent (Bio-Rad) was added to the protein solution in Milli-Q water and in concentration 30:1, as reported in the protocol provided by the supplier. The protein solutions have been measured by standard spectrophotometer technique as reference [6,12,33] by using a spectrophotometer (V-670 UV-VIS by Jasco Europe, Cremella, Italy) in order to measure their optical absorbance as a function of the wavelength and then analyzed by the opto-microfluidic device. A sketch of the reaction system is depicted in Figure 1a.

The opto-microfluidic device consists of a monolithic substrate of lithium niobate, where a cross-shaped droplet generator was engraved and coupled with an array of optical waveguides perpendicular to the main fluidic channel. While the former enables the droplets production inside the chip, the latter brings the integration of optical analysis stages that, otherwise, may require bulky instrumentation as spectrophotometers. By way of optical waveguides, in fact, laser light is confined and guided toward the microfluidic channel, thus illuminating the fluid flowing inside. These optical waveguides are z-propagating and have been realized on a 1-mm-thick commercial congruent LiNbO_3_ wafer (Crystal Technology) by titanium (Ti) local doping via thermal in-diffusion as already described by the authors in [34,35,36]. This preparation process was set up to obtain an array of 3 μm width optical waveguides, with a local increase of the refractive index in the doped region that assures the optical confinement by total internal reflection.

Single optical mode propagation within the waveguide was assessed by standard near-field techniques [35,37]. The waveguides array was then cut by a Disco saw 321 micro-machining, equipped with a diamond particle-polymeric blade, in order to engrave 200 μm wide and 100 μm deep microfluidic channels in a cross-configuration [37]. The chip edges and the lateral walls of the engraved circuit showed an excellent optical quality with a lateral surface roughness of about 4 nm RMS. The working principle of the opto-microfluidic device and its relative performance have already been discussed [36,37,38] and will not be treated here. Figure 1b reports a sketch of the opto-microfluidic platform together with its picture (Figure 1c), respectively.

In this configuration, the light exiting from the input waveguide interacts with what flows in front of it, droplet included. The output waveguide, being perfectly aligned to the input waveguide on the other side of the microfluidic channel, collects the transmitted light that is finally detected by a standard photodetector. The latter monitors the light intensity in real-time as well as any change due to its interaction with the flowing droplets.

The presented device was tested by droplets produced by injecting two immiscible fluids in a cross-flow junction configuration. Hexadecane oil (Sigma Aldrich, Merk Life Science S.r.l., Via Monte Rosa, 93, 20149 Milano, Italy; refractive index of 1.434 for wavelength of 632.8 nm) with 3% (*w*/*w*) concentration of SPAN80 (Sigma Aldrich) was flowed in the central channel with a flowrate of Q_C_ = 25 μL/min playing the role of the continuous phase. Milli-Q^®^ water or BSA protein solutions of different concentrations have been injected in the two orthogonal channels with flowrate Q_D_ varied between 2–10 μL/min, providing for the so-called dispersed phase (droplets). In particular, Milli-Q^®^ water was used to clean the two orthogonal channels as well as to generate water droplets to fully characterize the microfluidic chip performance.

The flow rates have been controlled by a pressure pump OB1 MK3 (Elveflow, Paris, France) in feedback with flowmeters BFS Coriolis (Bronkhorst, AK Ruurlo, The Netherlands). The optical setup exploited a He-Ne laser (632.8 nm, 1 mW laser) coupled to the input waveguide. A standard imaging setup video has recorded the droplets’ displacement inside the channel in a synchronous way to validate the performances of the opto-microfluidic device. The camera used is a Basler ACA800–510 um (Basler, Ahrensburg, Germany; 511 fps at maximum resolution 800 × 600) coupled with an objective lens (10×/0.25 Nikon). The analyses of the droplet videos, as well as the transmitted optical signal from the waveguide, were performed by means of customized software.

## 3. Results

**Figure 2:**
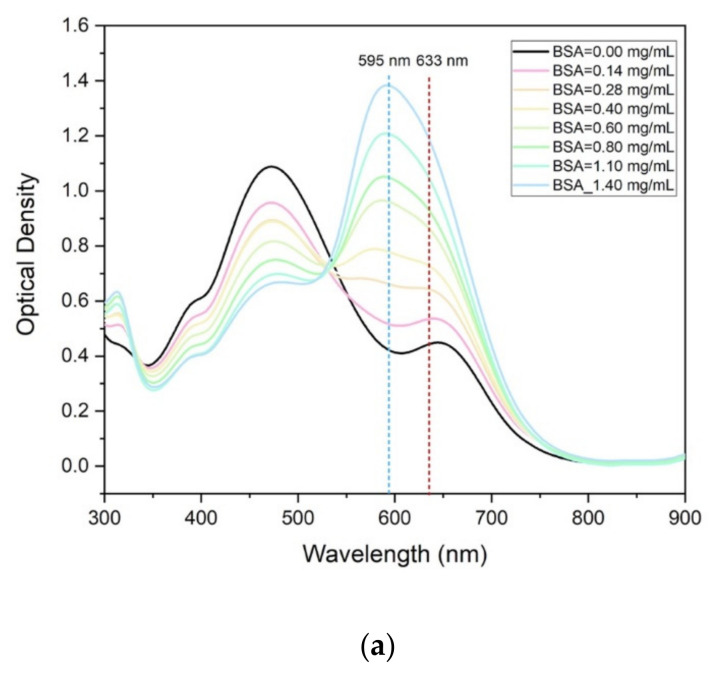

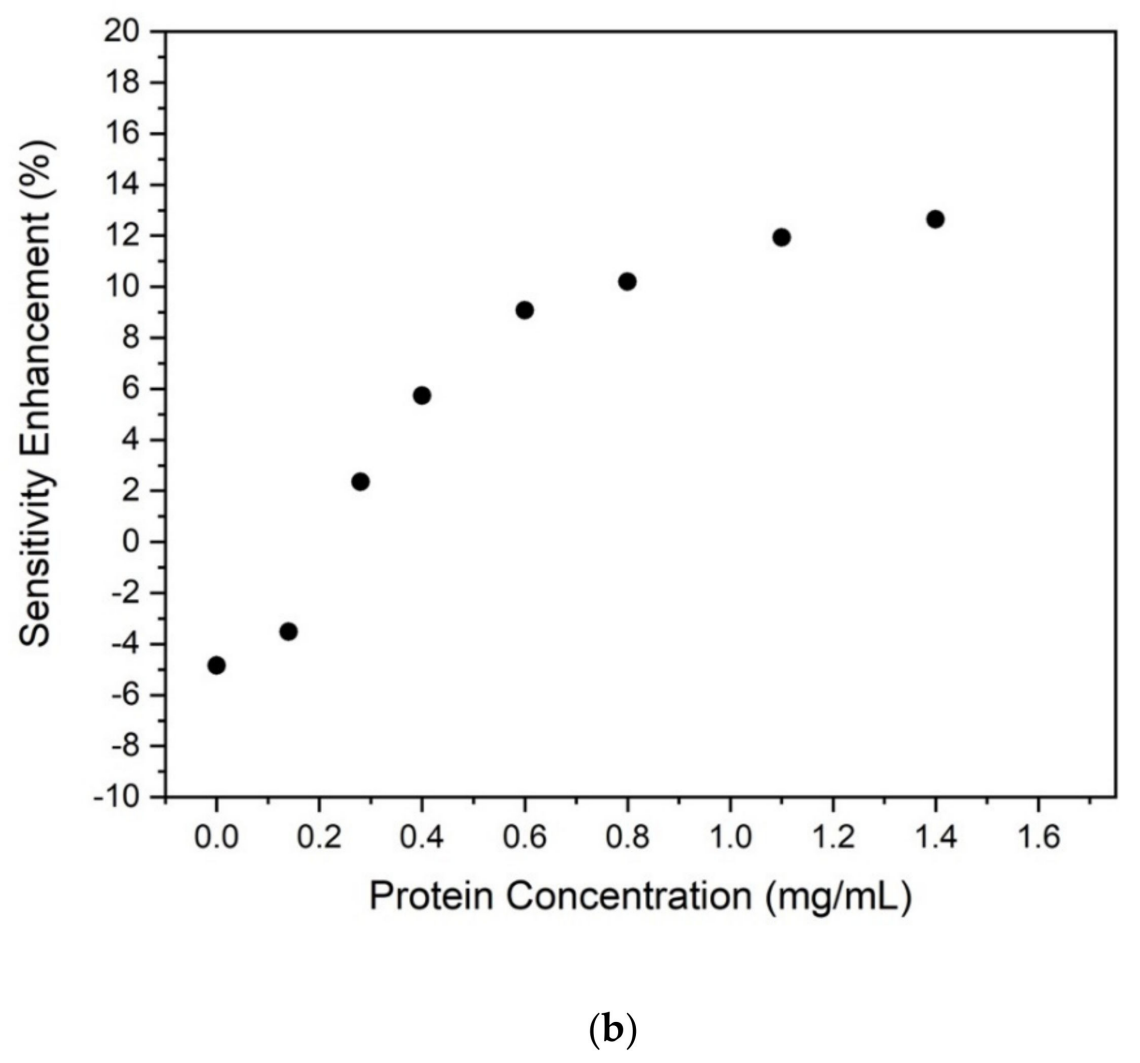
(**a**) Optical density of the protein solutions as a function of the wavelength (solution thickness = 1 cm). (**b**) Sensitivity Enhancement (SE) of OD at 633 nm with respect to OD at 595 nm as a function of the BSA protein concentration. SE = (1 − OD_633nm_/OD_595nm_)%.

The optical spectra collected at the spectrophotometer are reported in Figure 2a for various protein concentrations in the range 0–1.4 mg/mL compared to the sensitivity enhancement (SE) estimated as (1–OD_633nm_/OD_595nm_) in the function of the protein mass concentration (Figure 2b). In particular, OD_633nm_/OD_595nm_ is the Optical Density (OD) when measured at the peak 633 nm with respect to the peak at 595 nm, respectively.

For concentrations lower than 0.3 mg/mL, the sensitivity gained at 633 nm is slightly better than that achieved at 595 nm (SE is negative since OD_633nm_ > OD_595nm_) as the OD values and curves trends are close. On the contrary, for higher values of concentrations, the OD peak at 595 nm tends to increase faster than the 633 nm one, showing a maximum 12% gain with respect to the OD measured at 633 nm. It is evident that both remain suitable and reliable indicators to be used to estimate the protein concentration. This opens a wide perspective in the exploitation of standard and miniaturized diode laser light sources at 630–635 nm (commercially available at 25 Euro for the power of 5 mW), already integrated and integrable in microfluidic platforms, instead of the more expensive and bulky ones at 595 nm (still not commercially available as diode lasers). These results assess a high correlation of the light absorbance made at 595 nm is commonly used in the Bradford protein assay method with the one achieved at 633 nm. In the following discussion, the transmission peak at 633 nm will be therefore used as a working condition in the microfluidic device.

**Figure 3:**
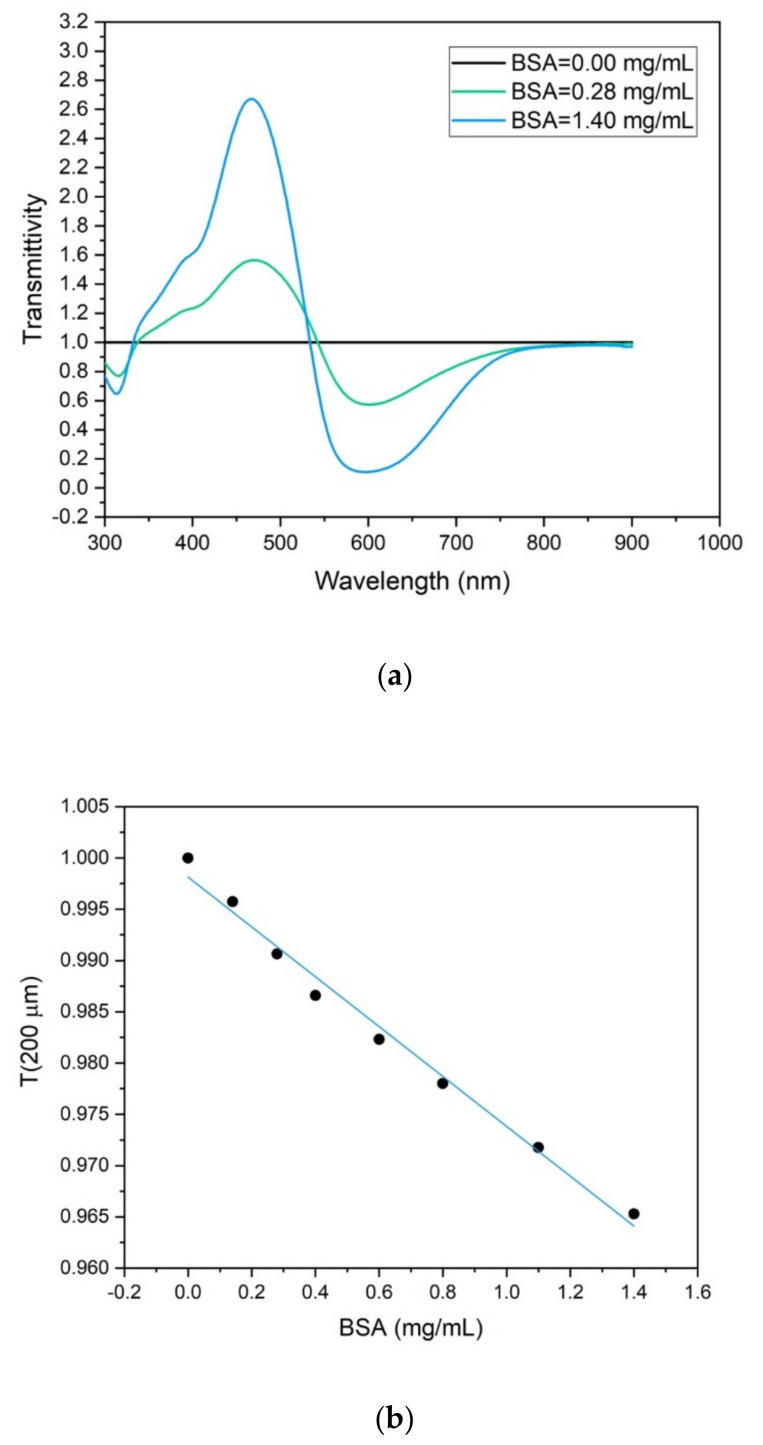
(**a**) Transmitted intensity of the protein solution with respect to the baseline, T = I_T,c_/I_T,BASE_ as a function of the wavelength for a solution thickness of 1 cm. (**b**) Transmitted intensity of the protein solution with respect to the baseline, T = I_T,c_/I_T,BASE_ as a function of the protein concentration for a solution thickness of 200 μm, acquisition at 633 nm.

In Figure 3a, the optical transmittance T is reported as a function of the wavelength λ, T is defined as the ratio of the transmitted intensity of the protein solution at concentration c (I_T,c_) with respect to that of the baseline (I_T,BASE_): T = T(λ) = I_T,c_/I_T,BASE_.

I_T,c_ and I_T,BASE_ can be easily derived by the optical absorption spectra obtained with the spectrophotometer, currently provided in terms of Optical density (OD)_._ For a proteins solution of concentration *c* OD is defined as $OD_{|c}=-{\mathrm{Log}}_{10}\left[\frac{I_{T,c}}{I_{O}}\right]$ where I_0_ is the transmitted intensity in an empty cuvette, with the baseline corresponding to c = 0. As a consequence, it results that $T=10^{-\left(OD_{|c}-OD_{|c=0}\right)}.$

In standard spectrophotometers, the optical path z equals the thickness of the cuvette used for the measurements (in this paper, 1 cm). From these data, it is possible to derive the transmitted light intensity corresponding to a solution thickness of 200 μm, i.e., equivalent to the absorbing thickness expected in the optofluidic device used in this work when the light, exiting from the optical waveguide, crosses the droplet in the microfluidic channel. In particular, it results that: $T_{|z=200\;\mu m}=T_{|z=1\;cm}{}^{\frac{200\;\mu m}{1\;cm}}$.

The results are reported in Figure 3b, where the transmitted light intensity at 632.8 nm was reported as a function of the protein concentration in the solution. The linear dependence therein showed suggests that only three different protein concentrations are needed for calibrating the device. In order to achieve accuracy better than 0.5%, the best choices correspond to the concentration values at the range extremes and one within the same range. In particular, in this paper, the following three concentrations have been consequently chosen for the device calibration: 0, 0.28, and 1.4 mg/mL, respectively. It is worth mentioning, however, that any concentration in the explored range [0; 1.4 mg/mL] can be taken as a reference for the calibration because of the linear dependence between transmission and concentration, as shown in Figure 3b.

**Figure 4:**
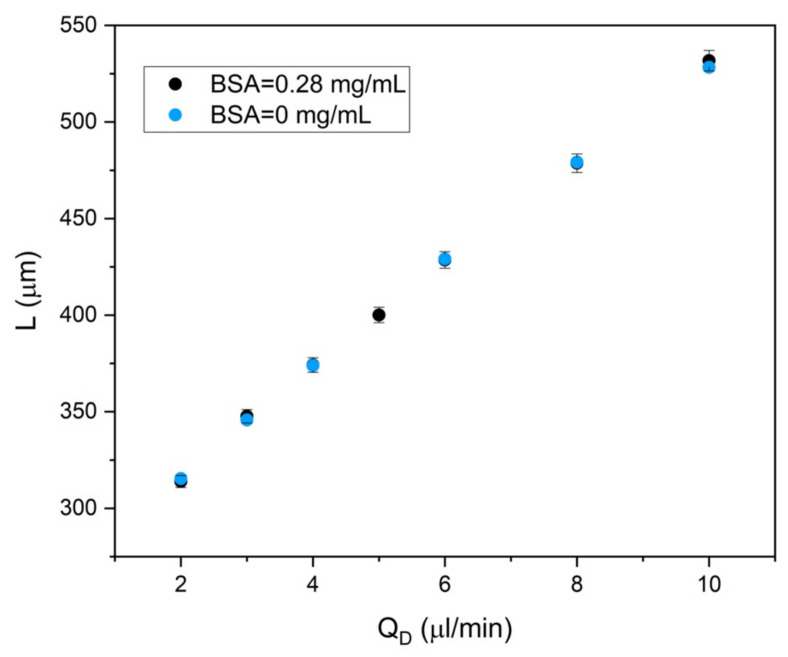
Droplet length as a function of dispersed phase flux Q_D_ at Q_C_ = 25 μL/min for concentrations of 0.28 and 0.0 mg/mL, respectively. The droplet length was measured by the camera and imaging processing as described in [34,35,36]. The same trend was observed when plotting the droplet velocity as a function of Q_D_, not shown here.

The same protein solutions have been therefore characterized in the opto-microfluidic platform in a droplet-based configuration. Droplets of different lengths were generated for every protein concentration under reproducible conditions of dispersed/continuous fluxes (Q_D_/Q_C_). Figure 4 reports an example of the typical linear dependence of the droplet length as a function of Q_D_ at Qc = 25 μL/min for solutions without protein (baseline) and with a protein concentration of 0.28 mg/mL (ratio 1:5 with respect to the maximum protein concentration used in this work). The linear trend is in good agreement with the theoretical and experimental observations, since droplets are generated in the so-called squeezing regime, in which droplets are squeezed in the channel and therefore, their length depends linearly on Q_D_/Q_C_ [38,39]. Moreover, Figure 4 shows that in this experimental configuration, it is possible to generate droplets of the same shape (i.e., length and velocity) and different content.

**Figure 5:**
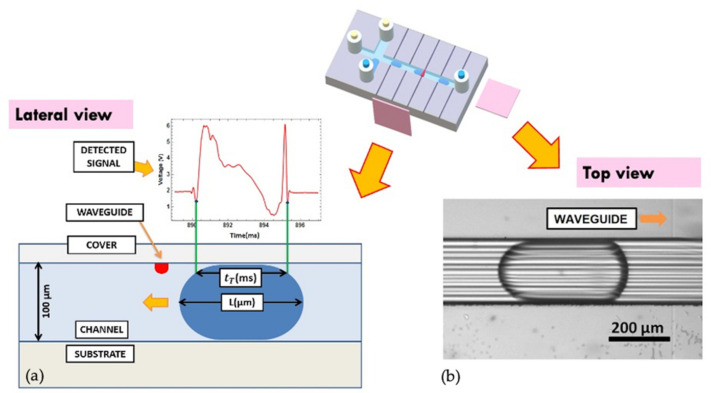
(**a**) Lateral view of the opto-microfluidic platform when the droplet passes in front of the waveguide. The transmitted intensity (detected signal) is recorded as a function of time: when the droplet passes in front of the waveguide, the transmitted intensity varies accordingly [34,35,36]. (**b**) Top view: camera picture of the droplet flowing into the microfluidic channel, approaching the optical waveguide (on the right of the picture). The pink squares identify the view planes with respect to the opto-microfluidic platform.

In fact, by a synchronous recording of the transmitted light intensity with the photodetector across each droplet and the relative videos from a top view, the camera acquisition of the droplet length was correlated with the transit time t_T_ of the droplet in front of the optical waveguide, cross-checking the stability of experimental conditions [34,35,36,37]. Although the transmitted light signal can result in a complicated time profile as a consequence of the droplet passage in front of the optical waveguide, it exhibits extremely reproducible features and univocally identified fingerprints [35]. This is very remarkable since the time evolution of the transmitted signal contains information on both the composition and the geometrical shape (length and velocity of the droplet included), since it is the convolution of transmitted, reflected, and diffracted beams from all the interfaces, respectively. They are clearly assigned to each droplet so that the camera acquisition and imaging processing are definitively not needed as demonstrated in [36]. An example of these fingerprints. as well as the relative physical quantities under consideration are shown in Figure 5a, where the transit time of passage t_T_ (in ms) is correlated to the physical droplet length L as measured by the camera (in in μm). Figure 5b reports a picture (top view) of the droplet flowing into the microfluidic channel, in the proximity of the optical waveguide. The relation between L and the length detected by the optical waveguide L_opt_ = vt_T_, with v being the droplet velocity, was evidenced. L is systematically higher than L_opt_ = vt_T_ because the light from the waveguide doesn’t intercept the droplet at its main axis but in its upper part by the geometry of the opto-microfluidic platform. This geometry allows the sensibility of the droplet shape to be dramatically enhanced and increases the detection capability of the system by identifying different droplets without impacting on the overall transmitted optical intensity detection. This is a key feature when background subtraction must be performed.

Despite the complex mechanisms of light–droplet interactions occurring when the light exiting from the input wavelength impinges on the droplet, the transmitted light intensity provides an extremely reproducible signal over hours provided that the droplets generation is made under controlled conditions in the same duration period. In fact, the fluctuation on droplet size distribution is due to the experimental variation of droplets generation since the opto-microfluidic platforms are made of a chemical-resistant substrate. In practice, fluctuations of the experimental conditions in the fluids’ injection (either by syringe pumps or by fluxmeters controlled in pressure of flux) are easily solved and do not constitute a limiting factor. As a matter of fact, the passage of one single droplet (dispersed phase) normally lasts from a few milliseconds up to ten milliseconds each and is alternated by the continuous phase flux that provides a few milliseconds plateau in the transmitted light. Consequently, in 1 min of data acquisition, an ensemble of 3000–15000 droplets was scanned, providing a reliable sample of reference for quantitative analysis that assumes already a statistical relevance. A flux stability in a period of time of at least one order of magnitude longer (ten minutes) guarantees minimized size dispersion (lower than 0.1%) in the produced droplets as huge as 30,000–150,000 units. In general, it is worth mentioning that it is always possible to generate a series of baseline/protein solution/baseline-droplets so that the comparison of the protein solution with the reference baseline can be carried out one-to-one in a time frame of reference that is not affected by changes in the experimental conditions, including temperature fluctuations.

**Figure 6:**
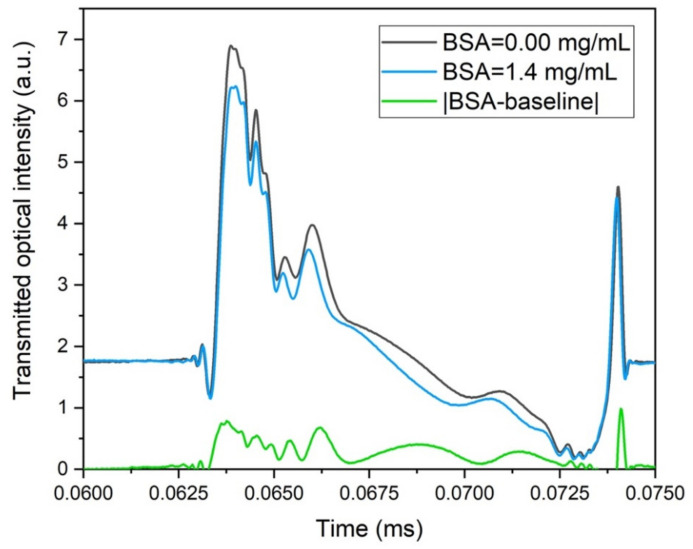
Example of the transmitted optical intensity detected by the opto-microfluidic platform of a droplet made of the protein solution (blue); the baseline-made droplet (black) and the absolute value of the difference signal resulting from subtracting the contribute of the baseline to protein solution transmission (green line). Protein concentration = 1.4 mg/mL, Q_D_ = 5 µL/min, Q_C_ = 25 µL/min, L = 401 ± 7 µm. Laser He-Ne wavelength: 632.8 nm.

By fixing the droplet generation parameters in order to have both the baseline and protein solution droplets with the same shape, it was therefore straightforward to provide for quantification of the protein content by subtracting the baseline contribute to the optical transmission of the protein solution signal on a single droplet. In Figure 6, an example of the transmitted optical intensity detected by the opto-microfluidic platform was presented in the case of protein concentrations equal to 1.4 mg/mL for: (i) droplet made of the protein solution (blue); (ii) baseline droplet (black); (iii) the difference signal resulting from subtracting the contribution of the baseline to the protein solution transmission (green line). Since at 632.8 nm, the absorbance of the protein solution is higher than the baseline, it was clearly distinguishable that the reduced transmission could be observed and the contribution of the protein content can be easily isolated.

The acquisition time can be reduced to less than one minute. Software routines to identify the droplet signal are extremely fast and straightforward since the beginning and the end of the droplet–light interaction can be set to the first minimum before and after the two maxima closest to the transmission plateau of the continuous phase. Similarly, the same routines provide the subtraction of the protein signal and the baseline ones in a separate output file, including its integral is a quantification of the overall protein content detected in the droplet. By the calibration curves of protein content vs. this integral, any type of droplet can be easily processed in a fully automatized protocol and in less than 2 min with the protein solution droplet (doped with the dye) still processable and/or reusable. In this work, three different concentrations have been used to create the calibration curve (namely 0, 0.28, and 1.4 mg/mL, respectively), and other concentrations were treated as tests to verify the accuracy of the predicted value with respect to the measured one, resulting in an accuracy better than (98 ± 1)%. Moreover, it is worth mentioning that the minimum amount of solution that is therefore needed is equal to the droplet volume, i.e., of the order of 3–10 nL in case of a microchannel of 200 μm × 100 μm and droplet length in the range 200–500 μm. It is equivalent to protein consumption in the range of 1.4 ng for a protein concentration of 1.4 mg/mL. 

In terms of the best values published in the literature [6], the opto-microfluidic approach for protein quantification presented here uses 1000 times less volume. It was measured a LOQ of at least 0.12 mg/mL, i.e., 10 times better than those reported in [6]). The observed LOD value is four times lower than the last literature results [6]. The high sensitivity of this opto-microfluidic platform to identify different droplets in a sequence of randomly generated ones, differing either for shape or composition or both, was already investigated in detail as reported in and will not be discussed further in this work. However, it is important to underline that this opto-microfluidic platform is able to detect and distinguish one single droplet in a sequence of others by analysing the light transmitted across the microfluidic channel without any need of complementary techniques.

The same opto-microfluidic platform can also be exploited in a certified quantification protocol where statistics checks are mandatory and false-positive rejections are needed. In this case. a sequence of droplets is used, with a number of droplets that can be as long as desired. For the protein quantification, it is suitable to exploit the average integral (*Int*) of the optical transmitted light signal of the droplets and to analyse trains of subsequent running droplets coming from the same protein solution batch. The number of droplets composing the batch samples can be of the order of magnitude of 100,000 in less than 10 min acquisition. In this case, the precision is better than 0.1%, i.e., better than 0.3% observed in the case of 1 min of acquisition. As self-calibration, *Int* can be normalized to: (i) the plateau value of the continuous phase transmission ($\bar{I_{c}}$) to compensate any effect due to the experimental condition instabilities (such as laser power fluctuations, temperature variations, etc.) and (ii) the droplet transit time, in order to obtain the mean droplet signal. As a consequence, the normalised *Int_n_* integral is achieved:

(1) $$Int_{n}=\frac{Int}{t_{T}}=\frac{\int_{t_{start}}^{t_{end}}\frac{I_{d}\left(t\right)}{\bar{I_{c}}}dt}{t_{end}-t_{start}}$$

**Figure 7:**
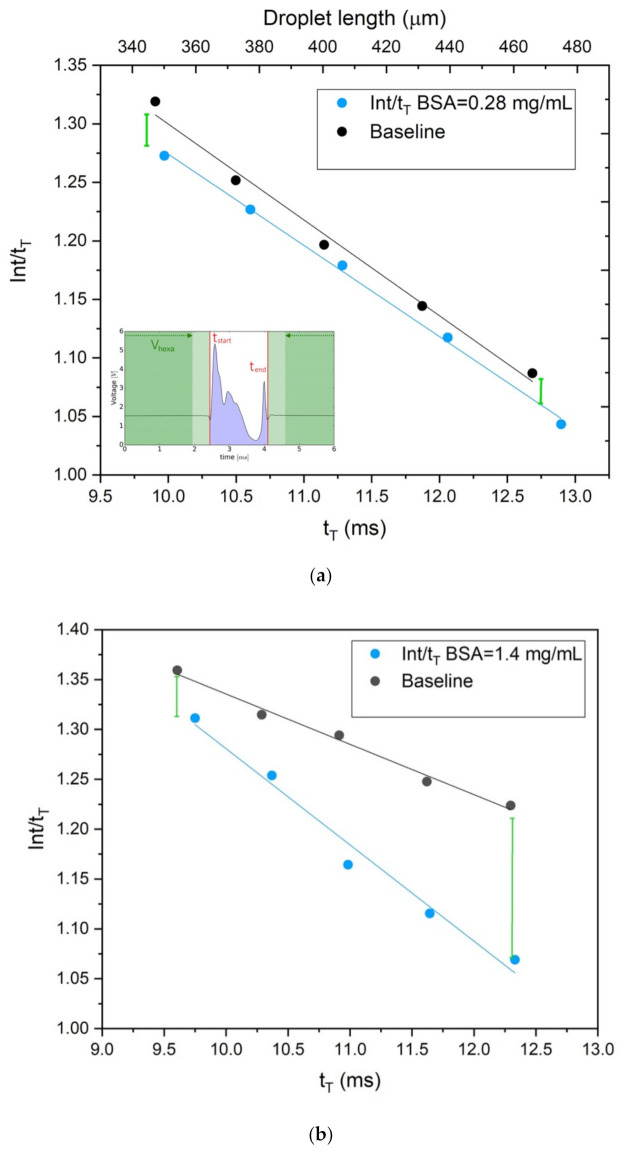
(**a**) Normalized integral Int/t_T_ of the optical intensity of the droplet (blue area in the inset) in function of t_T_. Protein concentration 0.28 mg/mL at Q_C_ = 25 μL/min. The green bars sketch the minimum/maximum detectable difference between the baseline droplet (BSA = 0.00 mg/mL) and the protein solution droplet respectively. (**b**) Normalized integral *Int/t_T_* of the optical intensity of the droplet (blue area in the inset) in function of t_T_. BSA Protein concentration 1.4 mg/mL at Q_C_ = 25 μL/min. The green bars sketch the minimum/maximum detectable difference between the baseline droplet (BSA = 0.00 mg/mL) and the protein solution droplet.

In Figure 7, we show *Int/t_T_* of the droplet (blue area in the inset of Figure 7a) as a function of *t_T_* for two different protein concentrations at a fixed Q_C_ value: (a) 0.28 mg/mL and (b) 1.4 mg/mL, respectively. As a matter of fact, *Int/t_T_* represents an indicator of the average detected transmissivity of the droplet, whilst the droplet duration *t_T_* (i.e., the physical length L of the droplet) can be varied by changing the ratio Q_D_/Q_C_. Calibration curves can be easily built and used for estimating the protein concentration at any Q_D_/Q_C_ value within the calibrated range. In particular, Figure 7 shows that the sensitivity given by this detection approach is shape-dependent and that the longer the droplet is, the better the sensitivity. Therefore, at a fixed Q_C_, it is possible to control the sensitivity by tuning the value of Q_D_ (and subsequently of *t*_*T*_).

In Table 1, the slope *m* and intercept *q* of the linear fits relative to the dependence *Int/t_T_* vs. *t_T_* are presented for some different BSA concentrations as explicative examples at a given microfluidics condition (in this case Q_C_ = 25 µL/min). The ratio *R = Int_n,Protein_/Int_n,Baseline_* of the integral made over the protein solution droplets and the relative baseline droplets was specified as well in Table 2. In fact, R is linearly dependent on the concentration of the protein solutions used in generating the droplets and, therefore, it provides direct info about the protein content. In the worst case, i.e., when the droplet shape is not optimized to get the maximum achievable sensitivity, R calibration curve evidences a LOQ equal to 0.06 mg/mL.

Once the droplet production parameters Q_C_ and Q_D_ (so that the droplet shape is fixed) were chosen, it was therefore possible to estimate the protein content by exploiting a standardized and automatized routine made of three steps:

(1) generation of a train of N baseline droplets and estimation of the mean parameter Int/t_T_ from the droplet signals: *Int_n,baseline_*;
(2) generation of a train of N protein solution droplets and estimation of the mean parameter *Int/t_T_* from the droplet signals: *Int_n,Protein_*;
(3) calculation of *R = Int_n,Protein_/Int_n,Baseline_* value. The direct comparison with the calibration curve R vs. concentration obtained at the same Q_C_ and Q_D_ values provides the average protein concentration on a train of droplets.

In particular, N depends on the acquisition time of the measure: for N = 1000, the baseline/protein solution acquisition would last 20 s each in the worst case, with an overall consumption of protein solution lower than 3 μL.

The experimental investigation on the role of the droplet size showed that longer droplets allow evidence the protein contributes to the absorbance (lower transmissivity) respect the baseline at equivalent droplet length/shape. When the lowest solution consumption is the main target, smaller droplets can be used without renouncing to get a good sensitivity. By decreasing the droplet volume of a factor of 3, the sensitivity decreases by a factor of 10. However, the difference *|Int_n,protein_–Int_n,baseline_|* is 10 times greater than the experimental error and therefore clearly identified also in this case. It is worth mentioning that the correlation of all the measurements varies the following parameters:

— fixed Q_C_, Q_D_ varied in the range [2;12] μL/min, 5 acquisitions of 90 min taken in different times for each value of Q_D_;
— fixed Q_D_, Q_C_ varied in the range [25;50] μL/min, 5 acquisitions of 90 min taken in different times for each value of Q_C_.

These three different concentrations of the protein solution (including the baseline, i.e., 0 mg/mL) were enough to get a calibration curve in the range [0, 1.4 mg/mL], providing a precision better than 0.3%. As a matter of fact, the LOQ value was confirmed to be at least 0.06 mg/mL in both the linear fitting procedures independently of including the concentration equal to 0.14 mg/mL (the lowest used in this experimental work) into the fitting procedure. This performance is promising, especially if compared to the most known Bradford protein concentration assay kits [6,40], as reported in Table 3. Moreover, for further comparison, the performance of Bicinchoninic acid (BCA) assays is reported as well. The BCA protein assay is, in fact, a widely used method for colorimetric detection and quantitation of total protein in a solution based on copper-based protein assay. It exploits a different working principle of the Bradford assay investigated here; that is, instead, a colorimetric dye-based method.

It is worth mentioning that droplet microfluidics presents the advantage of being compatible with any protein solution, the droplet being isolated from the microfluidic channel by way of the continuous phase. Finally, the working principle of the opto-microfluidic platform can be used with other immiscible fluids and is therefore not limited to water-based solutions.

## 4. Conclusions

The quantification of protein within water-based solutions with lower detection limits and higher sensitivity by way of a Bradford protein assay method was efficiently implemented in a portable opto-microfluidic platform and tested in a protein concentration range lower than 1.4 mg/mL with added value that the solution can be re-used for subsequent analyses. An opto-microfluidic platform equipped with integrated waveguides to illuminate protein solution droplets was used to measure the optical transmission of the solution. As a test, the BSA protein was investigated to verify the performance of the protein quantification protocol implemented here. We assessed the high correlation of the light absorbance made at 595 nm as commonly used in Bradford protein assay method with the one achieved at 632.8 nm as working conditions, i.e., at the emission wavelength of cheap and miniaturized diode lasers. The results demonstrated the possibility to quantify protein by using one single droplet, i.e., the volume of the order of nL, i.e., 1000 times less than the standard technique such as PADs-based ones. We measured a LOQ of at least 0.12 mg/mL, i.e., 10 times better than the standard procedures with PAD and achievable LOQ of 0.06 mg/mL. An observed LOD value that is four times lower than the last literature results was demonstrated with an accuracy of at least 98%.

## Figures and Tables

**Table 1:** Fit parameters of the fitting curves reported in Figure 7. Fitting line: Y = mX + q, Y = *Int*/*t_T_*, X = *t_T_* for some different BSA concentrations. Experimental conditions: Q_C_ = 25 µL/min.

Protein Concentration (mg/mL)	Slope m (ms^−1^)	Intercept q	Pearson’s r	R^2^
Baseline @ 0.28	−0.082 ± 0.005	2.19 ± 0.05	−0.995	0.990
Baseline @ 1.40	−0.050 ± 0.003	1.84 ± 0.09	−0.995	0.989
0.28	−0.050 ± 0.003	1.84 ± 0.09	−0.999	0.998
1.40	−0.097 ± 0.008	2.25±0.09	−0.990	0.980

**Table 2:** *R* as a function of BSA protein concentration C_BSA_, with *R = Int_n,Protein_*/*Int_n,baseline_*. (*) concentration used as test. Experimental conditions: Q_C_ = 25 µL/min, Q_D_ = 10 µL/min.

Protein Concentration (mg/mL)	*R* = *Int_n,Protein_*/*Int_n,baseline_*	1 − *R*
0.00	1.000 ± 0.005	0
0.14 *	0.993 ± 0.005 *	0.007 *
0.28	0.982 ± 0.004	0.018
1.40	0.892 ± 0.006	0.108

**Table 3:** Performances of the most known PAD-based protein concentration assay kits tested on BSA protein. For comparison, the Bradford assay (PAD in Microfiber) is compared to the Bicinchoninic acid (BCA) assay (PAD in cellulose) as well as to commercially available assay kits. (*) only incubation time. References: [a] ThermoFisher Part No. 23200 [b] ThermoFisher Part No. 23236 [c] Pierce™ Rapid Gold BCA Protein Assay Kit; [d] ThermoFisher Traditional BCA kit. Kits [a]-[b]-[c]-[d] are given calibration curves that are not linear, experimental sampling and spline fitting are therefore needed.

	BSA Range (mg/mL)	Assay Time (min)	Reagent Volume (μL)	Measured LOQ (mg/mL)	R^2^	Configuration	Ref.
Bradford assay	0.0–1.4	1	0.01	0.12	0.998	Single droplet	This work
	0.0–1.4	2	3	0.06	0.998	1000 Droplets	This work
	0.1–3.0	14	10	1.5	0.967	PAD	[6]
	0.125–1.5	10 (*)	35	0.08	Not linear		[a]
	0.125–1.5	10 (*)	20	0.12	Not linear		[b]
	0.125–1.5	10 (*)	400	0.025	Not linear		[b]
	0.001–0.025	10 (*)	1700	0.0025	Not linear		[b]
BCA assay	0.097–100.0	8	10	1.2	0.954	PAD	[6]
	0.020–2.0	5 (*)	20	0.13	0.999		[c]
	0.020–2.0	30	25	0.12	0.998		[d]

## References

[1] Sardesai V.V. (2011). Introduction to Clinical Nutrition.

[2] Murphy K., Bloom S.R. (2006). Gut hormones and the regulation of energy homeostasis. Nature.

[3] Seibert E., Tracy T.S. (2014). Fundamentals of Enzyme Kinetics. Methods Mol. Biol..

[4] Capra J.D., Aneway C.A., Travers P., Walport M. (2001). Immunobiology: The Immune System in Health and Disease.

[5] Clark M.L., Kumar P., Saunders Ltd. (2009). Kumar & Clark’s Clinical Medicine.

[6] Pravin P., Shashank J., Basant G. (2020). Selection of appropriate protein assay method for a paper microfluidics platform. Pract. Lab. Med..

[7] Chang S.K., Zhang Y., Nielsen S.S. (2017). Protein analysis. Food Analysis.

[8] Lomonossoff G.P., D’Aoust M.-A. (2016). Plant-produced biopharmaceuticals: A case of technical developments driving clinical deployment. Science.

[9] Hesse A., Weller M.G. (2016). Protein Quantification by Derivatization-Free High-Performance Liquid Chromatography of Aromatic Amino Acids. J. Amino Acids.

[10] Parr M.K., Montacir O., Montacir H. (2016). Physicochemical characterization of biopharmaceuticals. J. Pharm. Biomed. Anal..

[11] Toraño J.S., Ramautar R., De Jong G. (2019). Advances in capillary electrophoresis for the life sciences. J. Chromatogr. B Anal. Technol. Biomed. Life Sci..

[12] Bradford M.M. (1976). A rapid and sensitive method for the quantitation of microgram quantities of protein utilizing the prin-ciple of protein-dye binding. Anal. Biochem..

[13] Li X., Ballerini D.R., Shen W. (2012). A perspective on paper-based microfluidics: Current status and future trends. Biomicrofluidics.

[14] Yang Y., Noviana E., Nguyen M.P., Geiss B.J., Dandy D.S., Henry C.S. (2017). Paper-Based Microfluidic Devices: Emerging Themes and Applications. Anal. Chem..

[15] Roda A., Michelini E., Zangheri M., Di Fusco M., Calabria D., Simoni P. (2016). Smartphone-based biosensors: A critical review and perspectives. Trac. Trends Anal. Chem..

[16] Lan W.-J., Maxwell E.J., Parolo C., Bwambok D.K., Subramaniam A.B., Whitesides G.M. (2013). Paper-based electroanalytical devices with an integrated, stable reference electrode. Lab Chip.

[17] Sechi D., Greer B., Johnson J., Hashemi N. (2013). Three-Dimensional Paper-Based Microfluidic Device for Assays of Protein and Glucose in Urine. Anal. Chem..

[18] Songjaroen T., Dungchai W., Chailapakul O., Henry C.S., Laiwattanapaisal W. (2012). Blood separation on microfluidic pa-per-based analytical devices. Lab Chip.

[19] Radosevich M., Burnouf T. (2010). Intravenous immunoglobulin G: Trends in production methods, quality control and quality assurance. Vox Sang..

[20] Raynal B., Lenormand P., Baron B., Hoos S., England P. (2014). Quality assessment and optimization of purified protein sam-ples: Why and how?. Microb. Cell Fact..

[21] Gornall A.G., Bardawill C.J., David M.M. (1949). Determination of serum proteins by means of the biuret reaction. J. Biol. Chem..

[22] Doumas B.T., Watson W.A., Biggs (1971). H.G. Albumin standards and the measurement of serum albumin with bromocresol green. Clin. Chim. Acta.

[23] Legler G., Müller-Platz C.M., Mentges-Hettkamp M., Pflieger G., Jülich E. (1985). On the chemical basis of the Lowry protein determination. Anal. Biochem..

[24] Smith P.K., Krohn R.I., Hermanson G.T., Mallia A.K., Gartner F.H., Provenzano M.D., Fujimoto E.K., Goeke N.M., Olson B.J., Klenk D.C. (1985). Measurement of protein using bicinchoninic acid. Anal. Biochem..

[25] Flores R. (1978). A rapid and reproducible assay for quantitative estimation of proteins using bromophenol blue. Anal. Biochem..

[26] Weng L., Spoonamore J.E. (2019). Droplet Microfluidics-Enabled High-Throughput Screening for Protein Engineering. Micromachines.

[27] Tsou P.-H., Chiang P.-H., Lin Z.-T., Yang H.-C., Song H.-L., Li B.-R. (2020). Rapid purification of lung cancer cells in pleural effusion through spiral microfluidic channels for diagnosis improvement. Lab Chip.

[28] Sridhar A., Kapoor A., Kumar P.S., Muthamilselvi P., Balasubramanian S., Dai-Viet Nguyen V. (2021). Lab-on-a-chip technologies for food safety, processing, and packaging applications: A review. Environ. Chem. Lett..

[29] Lin P.-H., Li B.-R. (2021). Passively driven microfluidic device with simple operation in the development of nanolitre droplet assay in nucleic acid detection. Sci. Rep..

[30] Akgönüllü S., Bakhshpour M., Pişkin A.K., Denizli A. (2021). Microfluidic Systems for Cancer Diagnosis and Applications. Micromachines.

[31] Fang X., Wei S., Kong J. (2014). Paper-based microfluidics with high resolution, cut on a glass fiber membrane for bioassays. Lab Chip.

[32] Volpetti F., Garcia-Cordero J., Maerkl S.J. (2015). A Microfluidic Platform for High-Throughput Multiplexed Protein Quantitation. PLoS ONE.

[33] Zamboni R., Zaltron A., Izzo E., Bottaro G., Ferraro D., Sada C. (2020). Opto-Microfluidic System for Absorbance Measurements in Lithium Niobate Device Applied to pH Measurements. Sensors.

[34] Bettella G., Pozza G., Kroesen S., Zamboni R., Baggio E., Montevecchi C., Zaltron A., Gauthier-Manuel L., Mistura G., Furlan C. (2017). Lithium niobate micromachining for the fabrication of microfluidic droplet generators. Micromachines.

[35] Bettella G., Zamboni R., Pozza G., Zaltron A., Montevecchi C., Pierno M., Mistura G., Sada C., Gauthier-Manuel L., Chauvet M. (2019). LiNbO_3_ integrated system for opto-microfluidic sensing. Sens. Actuators B Chem..

[36] Zamboni R., Zaltron A., Chauvet M., Sada C. (2021). Real-time precise microfluidic droplets label-sequencing combined in a velocity detection sensor. Sci. Rep..

[37] Zaltron A., Bettella G., Pozza G., Zamboni R., Ciampolillo M., Argiolas N., Sada C., Kroesen S., Esseling M., Denz C. (2015). Integrated optics on lithium niobate for sensing applications. Opt. Sens..

[38] Chen X., Glawdel T., Cui N., Ren C.L. (2015). Model of droplet generation in flow focusing generators operating in the squeezing regime. Microfluid. Nanofluid.

[39] Van Loo S., Stoukatch S., Kraft M., Gilet T. (2016). Droplet formation by squeezing in a microfluidic cross-junction. Microfluid. Nanofluidics.

[40] Overview of Protein Assays Methods. https://assets.thermofisher.com/TFS-Assets/LSG/manuals/MAN0017135_PierceRapidGoldBCAProteinAssayKit_UG.pdf.

